# RNA-Seq transcriptome profiling reveals distinct immune response landscapes to identifying inflammation-related diagnostic markers in latent endometrial tuberculosis

**DOI:** 10.1038/s41598-025-89483-2

**Published:** 2025-04-10

**Authors:** Bai Dai, Jing-ying Liu, De-Bang Li, Zhi-ming Wang, Xiu-juan Chen

**Affiliations:** 1Reproductive medicine center, the Affiliated Hospital of Inner Mongolia Medical University, Hohhot, P.R. China

**Keywords:** Latent endometrial tuberculosis, Inflammatory biomarkers, RNA-Seq, Immune Response, Diagnosis, Female infertility, Transcriptomic analysis, Computational biology and bioinformatics, Biomarkers, Medical research, Reproductive disorders

## Abstract

Latent Endometrial Tuberculosis (LETB) is a significant yet under-recognized cause of female infertility, particularly in TB-prevalent regions. Current diagnostic methods for LETB lack specificity, complicating early detection. Through RNA-Seq transcriptome profiling, we aimed to uncover distinct immune response landscapes and identify novel inflammation-related diagnostic markers for LETB. Our study included clinical diagnostics, histological examinations, and transcriptomic analyses comparing differentially expressed genes (DEGs) among control, LETB, and active TB groups. We identified seven candidate genes (IFI30, HCK, SPI1, IL1B, ITGB2, and FCGR2A) uniquely associated with LETB. Bioinformatic analyses revealed these genes’ significant roles in immune regulation, including leukocyte activation, cytokine signaling, and myeloid leukocyte-mediated immunity. Gene Set Enrichment Analysis (GSEA) confirmed their involvement in key immune pathways such as cytokine-cytokine receptor interaction and leukocyte transendothelial migration. Validation through qPCR and immunohistochemistry confirmed the differential expression of these biomarkers in LETB tissues. These findings provide new insights into LETB pathogenesis, suggesting potential biomarkers for enhanced early diagnosis and treatment, ultimately aiming to improve reproductive health outcomes for affected women.

## Introduction

Latent tuberculosis (TB) remains a pressing global health concern, with approximately 1.7 billion individuals worldwide harboring latent infection and at risk of progressing to active TB^1^. Among these, latent genital tuberculosis (LGTB) poses a significant threat to female reproductive health, frequently resulting in infertility. Studies estimate that LGTB accounts for 5–10% of infertility cases in regions with high TB prevalence^2^. A critical manifestation of LGTB is latent endometrial tuberculosis (LETB), which significantly compromises reproductive health by inflicting structural and functional damage to the endometrial tissue, a key component for successful implantation and pregnancy^3^. The tuberculosis bacilli impair the endometrial environment, undermining its ability to support normal reproductive processes. This underscores the necessity for early detection and intervention to mitigate the impact of LETB on fertility^4^.

The early diagnosis of LETB is crucial for the success of assisted reproductive technologies (ART) and the prevention of adverse outcomes, such as pregnancy loss. At reproductive medicine centers, couples with a history of TB-related infertility often turn to ART to achieve conception. However, before initiating treatment, it is critical to ascertain whether Mycobacterium tuberculosis has invaded the reproductive system, particularly the endometrium. LETB can persist in the body asymptomatically, posing a diagnostic challenge due to its latent nature. If undetected, LETB may transition to active TB during ART procedures, such as embryo transfer, leading to severe complications, including pregnancy loss. Early and accurate diagnosis is therefore essential not only to prevent such complications but also to ensure optimal reproductive outcomes in affected women.

Despite its clinical significance, diagnosing LETB remains challenging due to the lack of specific and sensitive diagnostic tools. Conventional methods, including microbiological cultures and histopathological examinations, often fail to reliably detect latent infections due to their limited sensitivity and specificity^5^. These diagnostic shortcomings highlight an urgent need for novel approaches capable of identifying LETB at an early stage. Recent advances in molecular techniques, such as RNA sequencing (RNA-Seq), offer promising opportunities to uncover distinct molecular and immunological markers associated with LETB. These approaches hold the potential to revolutionize LETB diagnostics by providing a more sensitive and comprehensive understanding of its underlying biology^6^.

The immune system plays a pivotal role in the pathogenesis of TB, including its latent forms such as LETB. Host immune responses to Mycobacterium tuberculosis are central to the disease’s progression, with inflammation serving as a hallmark feature of TB infection. Cytokine profiles, such as elevated levels of IFN-γ and TNF-α, have been shown to correlate with latent TB infections, reflecting the immune system’s attempts to control bacterial persistence^7^. Investigating the immune landscape of LETB may provide critical insights into its pathogenesis and identify inflammation-related markers that are essential for diagnosis^8^. The interaction between TB bacilli and the host immune system often generates unique immunological signatures, which can be exploited to develop specific diagnostic tools^9^.

This study leverages RNA-Seq transcriptome profiling to identify distinct immune response landscapes and inflammation-related diagnostic markers in LETB. By analyzing gene expression patterns in endometrial tissues affected by latent TB, this research aims to uncover specific biomarkers that can enable early and precise diagnosis. The study adopts a systematic approach, including careful sample selection, RNA sequencing, and bioinformatics analysis, to identify potential diagnostic targets. Ultimately, the goal is to improve diagnostic accuracy for LETB, thereby facilitating earlier detection and more effective treatment strategies. Findings from this study will contribute significantly to understanding LETB pathogenesis and may pave the way for novel therapeutic interventions, enhancing reproductive health outcomes in affected women.

## Materials and methods

### Ethics statement

This study was approved by the ethical committee of the Affiliated Hospital of Inner Mongolia Medical University. Written informed consent was obtained from all study participants. All experimental procedures described in this study were carried out in accordance with the protocols approved by the ethical committee of the Affiliated Hospital of Inner Mongolia Medical University. All experimental designs, data collection, and analysis processes were performed in accordance with the standards of science and ethics.

### Research sample

#### Retrospective analysis sample

Medical records of 425 infertile patients who underwent the first cycle of IVF fresh embryo transfer at the Reproductive Medicine Center of Inner Mongolia Medical University Affiliated Hospital from April 2017 to April 2019 were collected. Inclusion criteria were as follows: (1) age between 22 and 40 years old; (2) no clinical manifestations of active tuberculosis; (3) exclusion of active tuberculosis by imaging and endometrial histopathology biopsy examination; (4) transfer of high-quality embryos of grade I-II. Exclusion criteria were: (1) patients with endometrial lesions such as endometrial polyps, complex endometrial hyperplasia, atypical endometrial hyperplasia, endometrial cancer, etc.; (2) pelvic endometriosis, uterine adenomyosis; (3) ovarian insufficiency, AMH < 1.1ng/ml; number of eggs obtained from bilateral ovaries ≤ 3; (4) patients solely undergoing assisted reproduction due to male factors. Specific grouping is shown in Table 1.

**Table 1:** Retrospective analysis sample grouping.

CG (TB-IGRA-, *n* = 278)		LETB (TB-IGRA+, *n* = 147)	
**NIG**	**IG**	**NIG**	**IG**
161	117	84	63

Note: Control Group (CG); Latent Endometrial Tuberculosis group (LETB); Inflammatory group (IG, CD38-& CD138-); Non-Inflammatory (NIG, CD38+& CD138+) group; The current diagnostic criteria consider a positive Tuberculosis Interferon-Gamma Release Assay (TB-IGRA), indicative of LETB suspicion, and positivity for both CD38 and CD138, suggesting inflammation.

#### Transcriptome analysis sample

From April 2017 to April 2019, women aged 20 to 40 who underwent IVF treatment at the Reproductive Medicine Center of Inner Mongolia Medical University Affiliated Hospital due to tubal factors were selected. Prior to entering the IVF cycle, routine TB-IGRA testing was conducted. After obtaining informed consent for endometrial biopsy to exclude endometrial inflammatory lesions, endometrial biopsies were taken in the mid-luteal phase for pathological testing including CD38 and CD138. Additionally, a small amount of endometrial tissue was preserved in EP tubes and frozen at −80 °C. Specific grouping is shown in Table 2.

**Table 2:** Transcriptome analysis sample grouping.

CG (TB-IGRA-, *n* = 6)		LETB (TB-IGRA+, *n* = 6)		TB (TB-IGRA+, *n* = 3)
NIG	IG	NIG	IG	
3	3	3	3	3

Note: Control Group (CG); Latent Endometrial Tuberculosis group (LETB); Inflammatory group (IG); Non-Inflammatory (NIG) group; Tuberculosis group (TB); The current diagnostic criteria consider a positive Tuberculosis Interferon-Gamma Release Assay (TB-IGRA), indicative of LETB suspicion, and positivity for both CD38 and CD138, suggesting inflammation.

#### Validation analysis sample

From April 2017 to April 2019, women aged 20 to 40 who underwent IVF treatment at the Reproductive Medicine Center of Inner Mongolia Medical University Affiliated Hospital due to tubal factors were selected. Prior to entering the IVF cycle, routine TB-IGRA testing was conducted. After obtaining informed consent for endometrial biopsy to exclude endometrial inflammatory lesions, endometrial biopsies were taken in the mid-luteal phase for pathological testing including CD38 and CD138. Additionally, a small amount of endometrial tissue was preserved in EP tubes and frozen at −80 °C. Specific grouping is shown in Table 3.

**Table 3:** Validation analysis sample grouping.

CG (TB-IGRA-, *n* = 4)		LETB (TB-IGRA+, *n* = 4)		TB (TB-IGRA+, *n* = 4)
NIG	IG	NIG	IG	
2	2	2	2	2

Note: Control Group (CG); Latent Endometrial Tuberculosis group (LETB); Inflammatory group (IG); Non-Inflammatory (NIG) group; Tuberculosis group (TB); The current diagnostic criteria consider a positive Tuberculosis Interferon-Gamma Release Assay (TB-IGRA), indicative of LETB suspicion, and positivity for both CD38 and CD138, suggesting inflammation.

### ELISA for tuberculosis diagnosis

In this study, we employed the TB-IGRA kit for adjunctive tuberculosis diagnosis using an ex vivo ELISA method. After collecting 4 mL of heparinized whole blood from each participant, aliquots were distributed into negative control, positive control, and patient culture mediums. Incubation at 37 °C for 22 ± 2 h followed, with subsequent plasma extraction by centrifugation at 4000 rpm for 10 min. Plasma samples were then analyzed for IFN-γ levels per the kit’s instructions, adhering strictly to specified storage and operational conditions. This rigorous approach yielded critical data for tuberculosis diagnosis.

### HE staining and immunohistochemistry

Tissue specimens were fixed in 10% neutral buffered formalin, routinely dehydrated, cleared, embedded in paraffin, and sectioned at a thickness of 3 μm. Hematoxylin and eosin (HE) staining was performed for histopathological evaluation. Immunohistochemistry was conducted using the EnVision two-step method. Primary antibodies against CD38, CD138, SPL1, and IL1B were purchased from Abcam.

### Doppler ultrasound examination

The Mindray color Doppler ultrasound diagnostic instruments are utilized, with probe frequencies ranging from 3.5 to 9.0 MHz. Through abdominal or vaginal examination, the uterus and bilateral adnexa are explored. Following the routine examination procedure, multi-directional, multi-sectional, and multi-angular scanning is performed. The location and size of lesions are recorded, and evaluation includes internal and posterior echogenicity, morphology, boundaries, and relationships with surrounding structures. Lesions are subjected to color Doppler detection, and images are stored.

### RNA extraction

Total RNA was extracted from the tissue using TRIzol^®^ Reagent following the manufacturer’s instructions. Subsequently, RNA quality was assessed using the 5300 Bioanalyzer (Agilent) and quantified using the ND-2000 (NanoDrop Technologies). Only high-quality RNA samples (OD260/280 = 1.8 ~ 2.2, OD260/230 ≥ 2.0, RIN ≥ 6.5, 28 S:18 S ≥ 1.0, > 1 µg) were utilized for constructing the sequencing library.

### Library preparation and sequencing

RNA purification, reverse transcription, library construction, and sequencing were conducted at Shanghai Majorbio Bio-pharm Biotechnology Co., Ltd. (Shanghai, China) following the manufacturer’s instructions (Illumina, San Diego, CA). The uterine endometrial tissue RNA-seq transcriptome library was prepared using Illumina^®^Stranded mRNA Prep, Ligation from Illumina (San Diego, CA) with 1µg of total RNA. Initially, messenger RNA was isolated via the polyA selection method using oligo(dT) beads and subsequently fragmented using a fragmentation buffer. Next, double-stranded cDNA was synthesized employing the SuperScript double-stranded cDNA synthesis kit (Invitrogen, CA) with random hexamer primers (Illumina). The synthesized cDNA underwent end-repair, phosphorylation, and ‘A’ base addition according to Illumina’s library construction protocol. Libraries were size-selected for cDNA target fragments of 300 bp on 2% Low Range Ultra Agarose, followed by PCR amplification using Phusion DNA polymerase (NEB) for 15 PCR cycles. After quantification using Qubit 4.0, the paired-end RNA-seq sequencing library was sequenced using the NovaSeq 6000 sequencer (2 × 150 bp read length). The raw paired end reads were trimmed and quality controlled by fastp^10^with default parameters. Then clean reads were separately aligned to reference genome with orientation mode using HISAT^11^software. The mapped reads of each sample were assembled by StringTie^12^ in a reference-based approach (Table 4).

**Table 4:** The mRNA sequencing data statistics.

Sample		Raw reads	Raw bases	Clean reads	Clean bases	Error rate (%)	Q20 (%)	Q30 (%)	GC (%)
CG	NIG	47,350,426	7,149,914,326	46,067,584	6,758,894,916	0.0268	97.20	92.57	51.94
		54,298,556	8,199,081,956	52,713,700	7,623,789,224	0.0264	97.37	92.95	51.93
		56,356,580	8,509,843,580	54,883,286	7,950,335,261	0.0266	97.28	92.75	52.33
	IG	43,141,658	6,514,390,358	41,434,754	6,078,093,406	0.0267	97.24	92.74	51.37
		53,446,106	8,070,362,006	51,813,696	7,511,173,014	0.0263	97.40	93.03	52.27
		53,016,154	8,005,439,254	51,276,054	7,392,516,867	0.0264	97.37	92.97	52.10
LETB	NIG	48,829,076	7,373,190,476	47,420,486	6,915,852,500	0.0263	97.42	93.05	52.33
		51,965,434	7,846,780,534	49,786,886	7,289,164,008	0.0272	97.01	92.22	50.79
		46,722,478	7,055,094,178	45,312,204	6,627,150,568	0.0262	97.48	93.15	52.94
	IG	48,005,458	7,248,824,158	46,989,584	6,893,496,419	0.0269	97.19	92.49	51.79
		55,793,572	8,424,829,372	53,772,540	7,800,850,282	0.0266	97.27	92.73	52.11
		49,896,196	7,534,325,596	48,599,792	7,113,620,448	0.0266	97.28	92.73	51.74
TB		50,342,790	7,601,761,290	48,473,942	7,093,253,613	0.0265	97.31	92.80	50.46
		46,850,508	7,074,426,708	45,472,326	6,663,625,083	0.0272	97.07	92.27	51.94
		55,172,404	8,331,033,004	53,863,158	7,920,817,759	0.0265	97.34	92.85	51.88

Note: Control Group (CG); Latent Endometrial Tuberculosis group (LETB); Inflammatory group (IG); Non-Inflammatory (NIG) group; Tuberculosis group (TB); The current diagnostic criteria consider a positive Tuberculosis Interferon-Gamma Release Assay (TB-IGRA), indicative of LETB suspicion, and positivity for both CD38 and CD138, suggesting inflammation.

### Differential expression analysis and functional enrichment

To identify differentially expressed genes (DEGs) between two distinct samples, transcript expression levels were quantified using the transcripts per million reads (TPM) method. Gene abundances were quantified using RSEM^13^Differential expression analysis was conducted using either DESeq2 or DEGseq. DEGs meeting the criteria of |log2FC| ≥ 1 and FDR ≤ 0.05 (DESeq2)^14^or FDR ≤ 0.001 (DEGseq)^15^were considered significantly differentially expressed. Additionally, functional enrichment analysis, encompassing Gene Ontology (GO) and Kyoto Encyclopedia of Genes and Genomes (KEGG) pathways, was performed to identify DEGs significantly enriched in GO terms and metabolic pathways, with a Bonferroni-corrected P-value ≤ 0.05 compared to the whole-transcriptome background. GO functional enrichment and KEGG pathway analysis were conducted using Goatools and KOBAS^16–19^, respectively.

### Set enrichment analysis (GSEA)

In this study, we conducted single-gene gene set enrichment analysis (GSEA) utilizing R programming language and its packages, specifically ‘limma’ for differential gene expression analysis, ‘ggplot2’ and ‘pheatmap’ for data visualization, ‘clusterProfiler’ and ‘enrichplot’ for enrichment analysis against databases such as KEGG, ‘org.Hs.eg.db’ for gene identifier conversion, and ‘patchwork’ along with ‘gseaplot2’ for the visualization of the enrichment results. This approach allowed us to delve into the enrichment of the gene sets associated with the gene of interest within immune-related biological pathways.

### Quantitative real-time PCR (qPCR) analysis

In this study, we employed quantitative real-time polymerase chain reaction (qPCR) technology to perform relative quantification analysis of gene expression in tissue samples. The experimental workflow included RNA extraction (using the OmniPlant RNA Kit from Beijing Kangwei Century Biotechnology Co., Ltd.), reverse transcription (with the HiScript Q RT SuperMix for qPCR from Nanjing Novozymes Biotechnology Co., Ltd.), and relative quantification of target genes versus reference genes (β-actin and GAPDH) using the ChamQ SYBR Color qPCR Master Mix (also from Nanjing Novozymes Biotechnology Co., Ltd.). Amplification and data acquisition were conducted using the LineGene9600plus real-time quantitative PCR instrument (Hangzhou Bioer Technology Co., Ltd.). Primers were synthesized with PAGE purification by Shenggong Bioengineering Co., Ltd., ensuring specificity and efficiency (Table 5).

**Table 5:** List of qPCR primers required for gene validation.

Primer	Base Sequence (5’ to 3’)
ssIFI30-F	TGTGACCCTCTACTATGAAGCA
ssIFI31-R	GGCACTTGAACTCCCACC
ssHCK-F	GGAGGCAATACATTCTCAAA
ssHCK-R	ATACAGGGCAACCACGAT
ssSP1-F	CACTGGAGGTGTCTGACGG
ssSP1-R	TGCTTGGACGAGAACTGGAA
ssIL1B-F	CTCGCCAGTGAAATGAT
ssIL1B-R	AAGCCCTTGCTGTAGTG
ssITGB2-F	CCCTCACCCTGTGGCAAGT
ssITGB2-R	TGCTCCAGCGTGTAGGC
ssFCGR2A-F	CTCCATCCCACAAGCAA
ssFCGR2A-R	CAGTCGCAATGACCACA

### Statistical analysis

Statistical significance was determined by one-way ANOVA using SPSS software (SPSS 21.0 for Windows). All productionrelated data are reported as the mean ± SE, and differences were considered significant at *p* < 0.05.

## Results

### Absence of reliable diagnostic markers for latent endometrial tuberculosis (LETB)

**Fig. 1:**
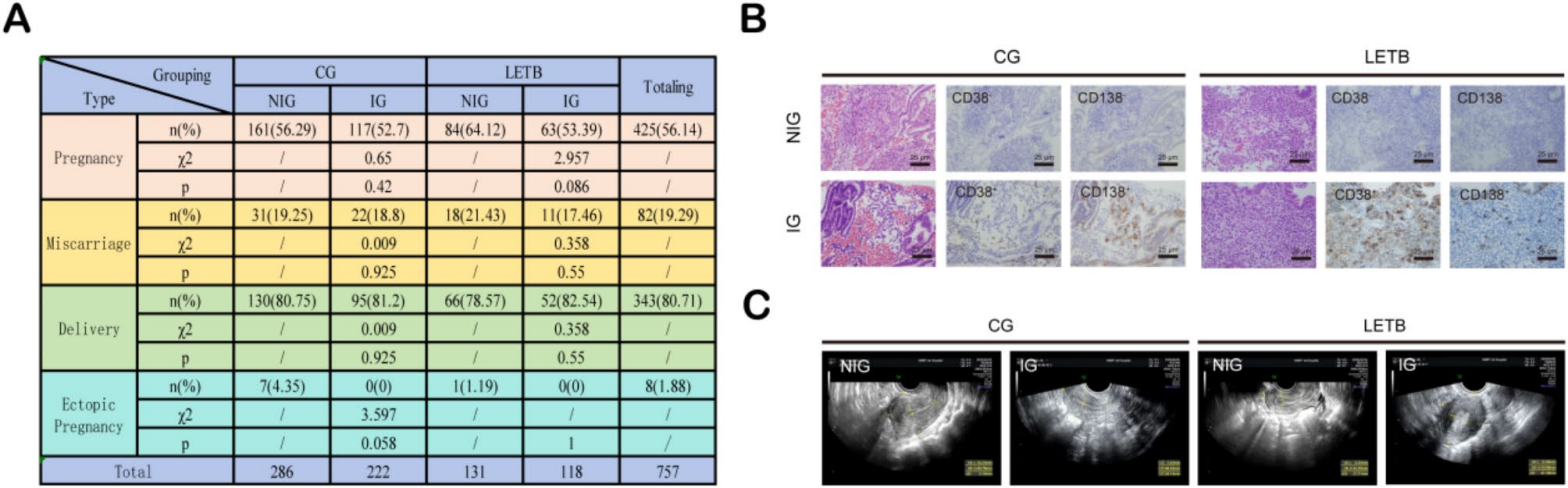
Lack of Reliable Diagnostic Markers for Latent Endometrial Tuberculosis (LETB). (**A**) Comparison of Pregnancy Outcomes between Control Group (CG) and LETB. The current diagnostic criteria consider a positive Tuberculosis Interferon-Gamma Release Assay (TB-IGRA), indicative of LETB suspicion, and positivity for both CD38 and CD138, suggesting inflammation. The study reveals no significant differences in pregnancy outcomes between the Inflammatory (IG) and Non-Inflammatory (NIG) groups (*P* > 0.05). (“/“ symbol indicates unavailable data). (**B**) Comparison of Hematoxylin and Eosin (HE) staining and Immunohistochemistry (IHC) results between CG and LETB. CD38 and CD138 serve as marker genes. Scale bar, 25 mm. (**C**) Comparison of intrauterine ultrasound images between CG and LETB. The morphology of the endometrial cavity shows uniformity without thinning of the endometrium (typically defined as < 7 mm). No significant differences were observed between CG and LETB in terms of endometrial morphology, thickness, and uterine cavity volume.

The endometrium often accompanies inflammation during the immune response against latent tuberculosis. Our study aimed to explore reliable inflammatory biomarkers for LETB diagnosis. Initially, we investigated pregnancy outcomes by comparing the Control Group (CG) and LETB, using the following criteria for grouping. Current diagnostic standards utilized positive Tuberculosis Interferon-Gamma Release Assay (TB-IGRA) results as indicative of LETB, along with positivity for CD38 and CD138, suggesting inflammation. The results revealed no significant differences in pregnancy outcomes between the Inflammatory (IG) and Non-Inflammatory (NIG) groups (*P* > 0.05), indicating the lack of specific diagnostic markers for LETB (Fig. 1A).

Subsequently, we compared Hematoxylin and Eosin (HE) staining results between the control group and LETB, showing no significant differences. Immunohistochemistry (IHC) using CD38 and CD138 as inflammatory markers also showed no significant difference in inflammation levels between LETB and the control group (Fig. 1B). These findings further support the conclusion that LETB lacks specific inflammatory markers. Additionally, comparison of intrauterine ultrasound images between the control group and LETB showed no significant differences in endometrial morphology, thickness, or uterine cavity volume. Overall, our experimental results indicate the absence of specific inflammatory markers associated with LETB (Fig. 1C).

### Transcriptomic analysis reveals novel inflammatory biomarkers for LETB

**Fig. 2:**
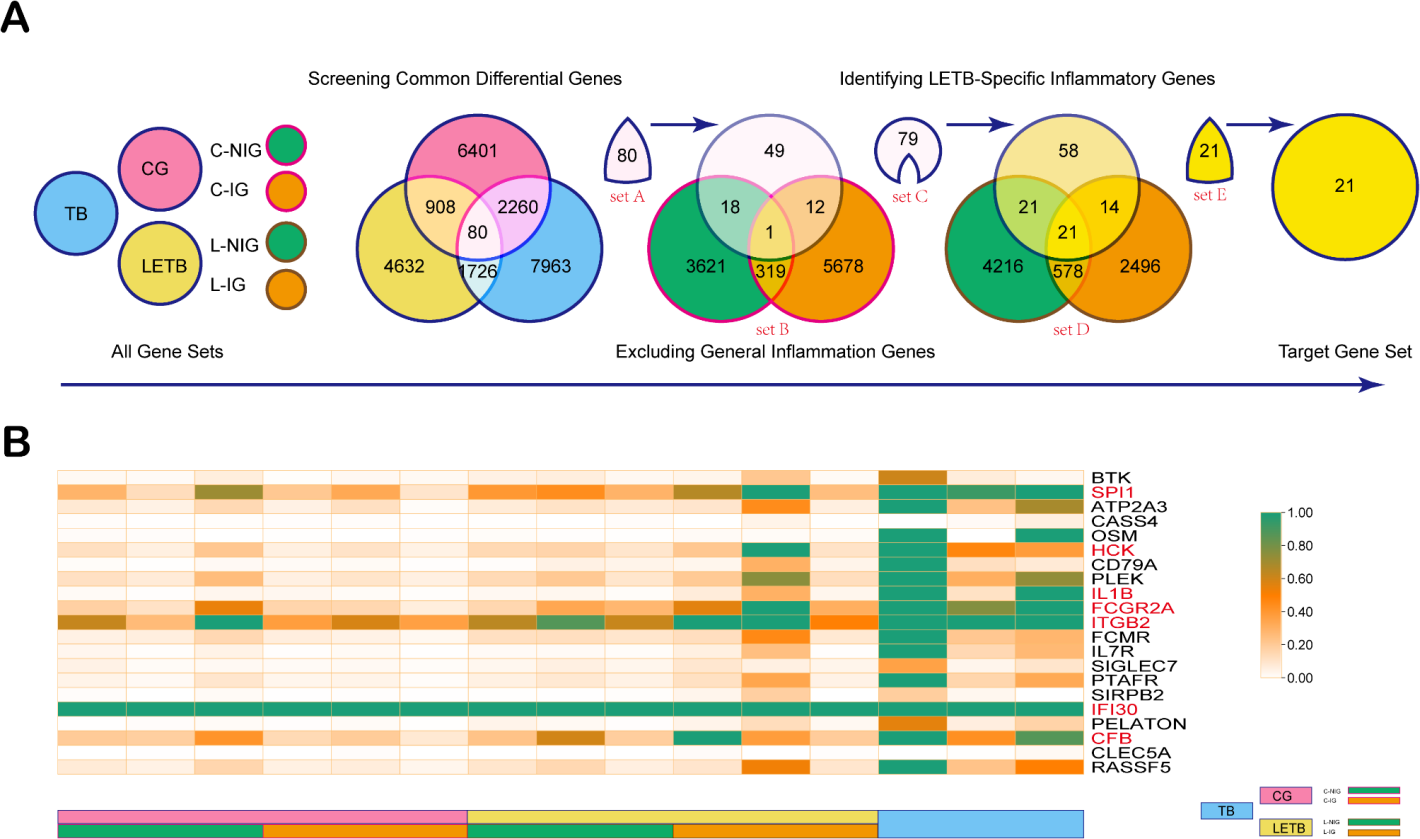
Transcriptomic Analysis Attempts to Identify Novel Inflammatory Biomarkers for LETB. (**A**) The Venn diagram illustrates the screening process of potential inflammatory biomarkers. Initially, intersections were taken between the control group (CG), latent endometrial tuberculosis group (LETB), and tuberculosis group (TB) pairwise, followed by a subsequent intersection of the three sets to obtain intersection A, representing the common differentially expressed gene set A, serving as the candidate genes for potential diagnostic targets. Subsequently, interference from common inflammatory genes was excluded from the candidate gene set A, resulting in gene set C. Specifically, intersections were taken between CG-NIG and CG-IG to obtain the intersection B of common inflammatory genes, followed by the removal of genes contained in B from gene set A to derive gene set C. Finally, a specific set of inflammatory genes induced by latent tuberculosis was identified from gene set C as the final candidate gene set E. The method involved taking intersections between LETB-NIG and LETB-IG to obtain intersection D, followed by taking the intersection of gene sets C and D to derive gene set E. (**B**) Heatmap illustrating the differential expression of genes in gene set E across the Control Group (CG), Latent Endometrial Tuberculosis Group (LETB), and Tuberculosis Group (TB). In this analysis, downregulated genes are shown in orange, while upregulated genes are depicted in green. Through preliminary screening of gene set E, seven candidate inflammatory biomarkers were identified and highlighted in red. This visualization highlights the distinct expression patterns of these potential diagnostic markers, emphasizing their relevance in the immune response associated with LETB.

Transcriptomic analysis aimed to uncover novel inflammatory biomarkers associated with latent endometrial tuberculosis (LETB). Through a systematic screening process, a series of pairwise intersections were performed among the control group (CG), LETB, and tuberculosis group (TB), followed by a subsequent intersection of the three sets. This approach yielded intersection A, representing a common pool of differentially expressed genes shared across the groups, thus serving as potential diagnostic targets. Further refinement involved excluding interference from common inflammatory genes. By intersecting CG-NIG and CG-IG, intersection B, comprising common inflammatory genes, was identified. Subsequently, genes in intersection B were removed from gene set A to derive gene set C, which specifically captured LETB-related inflammatory biomarkers. Lastly, intersections between LETB-NIG and LETB-IG revealed a specific set of inflammatory genes induced by latent tuberculosis, constituting the final candidate gene set E (Fig. 2A). Upon obtaining gene set E, we conducted an initial heatmap analysis to visualize the differential expression patterns of these genes across the CG, LETB, and TB groups (Fig. 1B). This preliminary screening facilitated the identification of seven candidate genes (IFI30, HCK, SPI1, IL1B, ITGB2, FCGR2A) based on their distinct expression profiles, statistical significance (p-value < 0.05), and functional relevance in immune regulation. The heatmap highlighted these genes in red, indicating their specific upregulation or downregulation in LETB tissues. The differential expression patterns of these potential biomarkers were visualized in a heatmap, showcasing downregulated genes in orange and upregulated genes in green across CG, LETB, and TB groups. Seven candidate genes, identified through rigorous screening, were highlighted in red (Fig. 2B).

### Unveiling distinct immune response functional enrichment and protein interaction networks in LETB-associated candidate genes

Building on the identification of candidate genes in gene set E (described in 3.2), we further explored their functional roles in LETB pathogenesis through enrichment analysis and protein interaction studies.

**Fig. 3:**
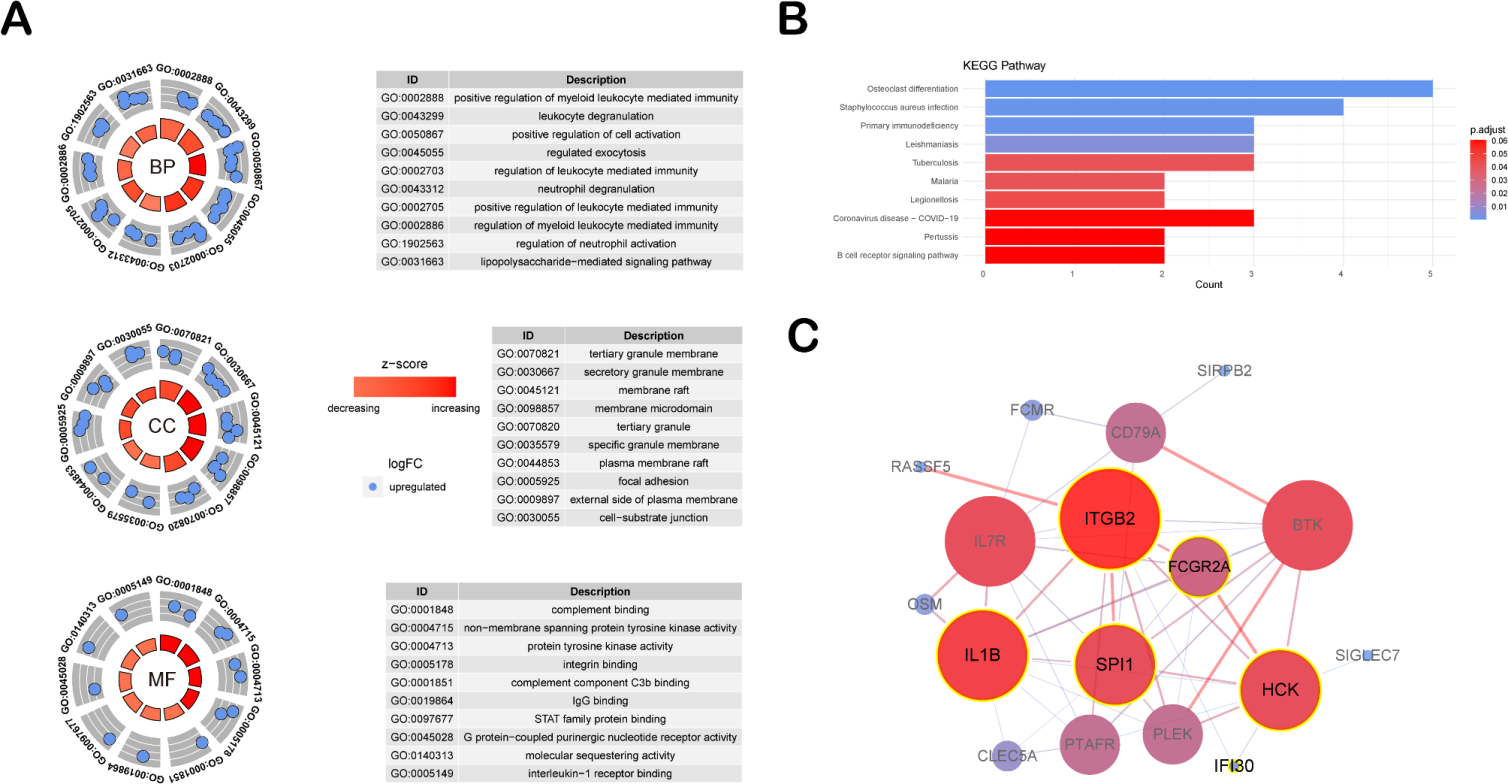
Functional Enrichment Analysis of Candidate Genes and Protein-Protein Interaction Network Exploration. (**A**) Gene Ontology (GO) analysis highlights the top ten terms in biological processes (BP), cellular components (CC), and molecular functions (MF) for a set of candidate genes derived from gene set E. Z-scores and log fold changes (logFC) quantify expression trends, with ‘upregulated’ indicating increased expression and ‘decreasing’/‘increasing’ reflecting changes in expression levels. (**B**) The overall KEGG pathway classifcation of the genes from gene set E. Top 10 up-regulated KEGG pathways are presented^17–19^. (**C**) For the candidate genes potentially indicative of LETB, protein-protein interaction (PPI) analysis was conducted using STRING (PPI enrichment p-value < 1.0e-16). Line thickness indicates the confidence level of interactions, with only high-confidence interactions displayed. The PPI network is enriched for immune response pathway genes (false discovery rate = 5.51e-06).

Gene Ontology (GO) analysis revealed that the seven candidate genes (IFI30, HCK, SPI1, IL1B, ITGB2, FCGR2A) are significantly enriched in biological processes associated with immune response regulation. Key processes include positive regulation of myeloid leukocyte-mediated immunity, leukocyte degranulation, and cell activation. Cellular component analysis highlighted their localization to immune-related structures, such as tertiary granule membranes, secretory granule membranes, and membrane rafts, suggesting roles in intracellular signaling and material transport. At the molecular function level, these genes were associated with complement binding, protein tyrosine kinase activity, and STAT family protein binding, highlighting their importance in immune signaling pathways (Fig. 3A).

KEGG pathway enrichment analysis demonstrated that these genes are significantly involved in LETB-relevant pathways, particularly cytokine-cytokine receptor interaction, leukocyte transendothelial migration, and NF-κB signaling. These pathways are critical in driving inflammation and immune responses in LETB, providing insights into the molecular mechanisms underpinning disease progression (Fig. 3B).

To further investigate the interactions and network relationships among these genes, we performed a protein-protein interaction (PPI) analysis. The analysis revealed that key proteins encoded by these genes, such as HCK, SPI1, IL1B, ITGB2, and FCGR2A, are central nodes in immune regulatory networks. Their interactions suggest a coordinated role in regulating immune activation and inflammation in LETB. These central nodes may represent potential therapeutic or diagnostic targets for LETB (Fig. 3C).

Integrating these findings, we propose that the candidate genes identified from gene set E not only reflect distinct immune response landscapes in LETB but also serve as critical mediators of immune regulation and inflammation. Their roles in key immune pathways underscore their potential utility as diagnostic biomarkers and therapeutic targets for LETB.

### Validating tuberculosis genomic markers: bridging transcriptomics with clinical application

In our study, we conducted a comprehensive analysis using Gene Set Enrichment Analysis (GSEA) to investigate the potential roles of six key genes (IF130, HCK, SPI1, IL1B, ITGB2, and FCGR2A) identified in endometrial tissues with latent tuberculosis infection, within immune-related biological pathways. Our analysis revealed significant enrichment of these genes in multiple pathways closely associated with immune response, including “KEGG_CYTOKINE_CYTOKINE_RECEPTOR_INTERACTION,” “KEGG_AXON_GUIDANCE,” “KEGG_LEUKOCYTE_TRANSENDOTHELIAL_MIGRATION,” and “KEGG_CHEMOKINE_SIGNALING_PATHWAY.”

**Fig. 4:**
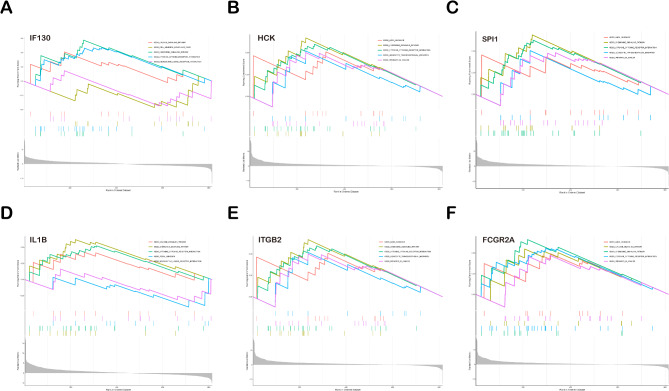
Gene set enrichment analysis of potential LETB biomarkers. Analyses of IFI30 (**A**) HCK (**B**) SPI1 (**C**) IL1B (**D**) ITGB2 (**E**) FCGR2A (**F**) were carried out. LETB, latent endometrial tuberculosis.

These pathways share a common theme in their core roles in communication, migration, and signal transduction among immune cells. Particularly, the enrichment of IF130, HCK, SPI1, IL1B, ITGB2, and FCGR2A in the “KEGG_CYTOKINE_CYTOKINE_RECEPTOR_INTERACTION” pathway suggests their potential involvement in regulating the interaction between cytokines and their receptors, a crucial step in immune response. Furthermore, the enrichment of ITGB2 in the “KEGG_LEUKOCYTE_TRANSENDOTHELIAL_MIGRATION” pathway implies its importance in the migration of immune cells across the endothelial layer, crucial for immune cell trafficking to sites of infection or inflammation. The enrichment of FCGR2A in the “KEGG_CHEMOKINE_SIGNALING_PATHWAY” pathway may be related to the chemotaxis and signaling mechanisms of immune cells (Fig. 4).

**Fig. 5:**
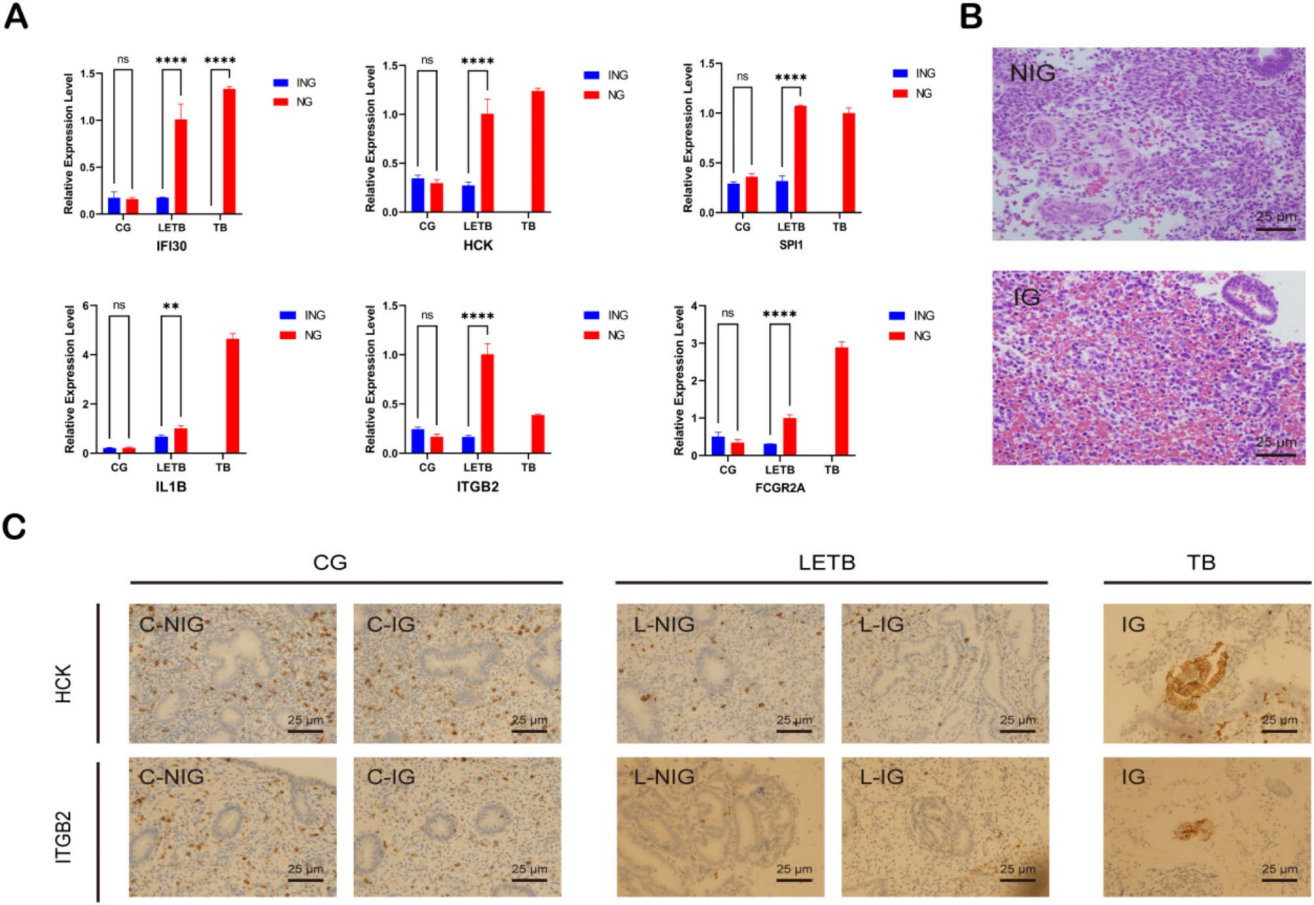
Validation of Potential LETB Biomarkers through Clinical Samples. (**A**) Validation of potential LETB biomarkers by qPCR; * indicates that the relative expression differs (*p* < 0.05). (**B**) Comparison of HE staining results between the Inflammatory (IG) and Non-Inflammatory (NIG) groups. (**C**) Validation of potential LETB biomarkers by Immunohistochemistry.

Based on the analysis, we validated potential LETB biomarkers through qPCR, HE staining, and immunohistochemistry. The qPCR results indicated that the relative expression of IFI30, HCK, SPI1, IL1B, ITGB2, and FCGR2A was significantly higher in the inflammatory group (IG) compared to the non-inflammatory group (NIG) (*p* < 0.05). HE staining revealed notable inflammatory cell infiltration and structural alterations in the IG, whereas the NIG maintained relatively normal tissue architecture. Additionally, based on qPCR data, we selected HCK and ITGB2 for immunohistochemical validation, which demonstrated significantly enhanced expression in the IG. These findings collectively suggest that these biomarkers hold substantial clinical diagnostic potential in inflammatory conditions (Fig. 5).

## Discussion

Our study aimed to identify specific biomarkers for Latent Endometrial Tuberculosis (LETB) by employing RNA-Seq transcriptome profiling. Initial comparisons revealed that conventional diagnostic methods, including TB-IGRA and markers like CD38 and CD138, were insufficient in distinguishing LETB from control samples based on pregnancy outcomes, histological examinations, and immunohistochemistry (IHC) results. Subsequent transcriptomic analyses identified seven candidate genes (IFI30, HCK, SPI1, IL1B, ITGB2, and FCGR2A) uniquely associated with LETB, which were further validated through quantitative PCR (qPCR) and IHC, confirming their differential expression in LETB tissues.

The immune response in LETB is characterized by unique inflammatory markers that differentiate it from other forms of tuberculosis and from healthy control endometrial tissues. Our study found that genes such as IFI30 and SPI1 play significant roles in myeloid leukocyte-mediated immunity and leukocyte degranulation, indicating a robust immune response specific to LETB. Gene Ontology (GO) and KEGG pathway analyses revealed these genes’ involvement in critical immune pathways, such as cytokine-cytokine receptor interaction and leukocyte transendothelial migration. These pathways are essential for immune cell communication, migration, and activation, which are crucial for mounting an effective response against latent infections.

Drawing inspiration from the studies of Rull et al.^20^. and Uuskula et al.^21^. our research has identified a set of genes associated with LETB that could serve as potential biomarkers. These findings align with the increased expression of TRAIL and S100A8 in recurrent miscarriage (RM) as noted by Rull et al., and they also echo the work of Uuskula et al. on placental gene expression dynamics in pregnancy complications. Our study contributes to the understanding of LETB and its impact on reproductive health, offering new avenues for early detection and management strategies that could improve outcomes for affected women.

The six genes identified (IFI30, HCK, SPI1, IL1B, ITGB2, and FCGR2A) have been implicated in various immune regulatory mechanisms and inflammation associated with tuberculosis. IFI30, known for its role in antigen processing and presentation, is critical in the immune system’s ability to recognize and respond to Mycobacterium tuberculosis^22,23^. HCK and SPI1 are involved in the signaling pathways that activate macrophages and other immune cells, highlighting their importance in controlling TB infection^22,23^. IL1B, a key pro-inflammatory cytokine, has been shown to be elevated in TB infections, contributing to the inflammatory milieu necessary for fighting the pathogen^24^. ITGB2 and FCGR2A are integral to leukocyte adhesion and migration, facilitating the movement of immune cells to sites of infection^25–28^.

In addition to their role in TB-related immune responses, these genes have been implicated in other health conditions involving inflammation. For instance, IFI30 has been linked to autoimmune diseases such as rheumatoid arthritis, where its antigen processing activity exacerbates immune dysregulation^29^. Similarly, HCK and SPI1 are known to regulate myeloid cell activation and cytokine signaling in chronic inflammatory conditions, including sepsis and inflammatory bowel disease^30^. IL1B is a well-characterized driver of systemic inflammation and has been associated with a wide range of pathological conditions, including atherosclerosis and obesity-related inflammation^31–33^. ITGB2, critical for leukocyte adhesion, has been implicated in immune deficiencies and aberrant inflammatory responses, while FCGR2A, an important mediator of phagocytosis, plays a key role in autoimmune diseases like systemic lupus erythematosus and rheumatoid arthritis^29,31^. These connections highlight the broader biological relevance of the genes identified in our study and provide insights into their potential roles in systemic immune responses.

Furthermore, our findings may have implications beyond TB, as inflammatory mechanisms similar to those observed in LETB are also seen in pregnancy pathologies such as recurrent miscarriage and preeclampsia. For example, ITGB2 has been implicated in improper trophoblast invasion and placental dysfunction^21,34–36^, while IL1B is known to play a role in pregnancy-related inflammatory disorders, including preterm labor and pregnancy loss^20,32,33,36,37^. These parallels suggest that the immune dysregulation identified in LETB may also have relevance to other inflammatory conditions impacting reproductive health.

In this study, we employed RNA-Seq technology to conduct a comprehensive analysis of the immune response characteristics associated with Latent Endometrial Tuberculosis (LETB), successfully identifying inflammation-related genes specific to LETB. Compared to the genome-wide expression analysis method utilized^21^, RNA-Seq offers enhanced resolution, allowing us to more precisely capture gene expression variations specific to LETB. Furthermore, while Uuskula et al. also applied transcriptome analysis to study the dynamic changes in placental gene expression, our research focused on the immune response to LETB, further demonstrating the broad applicability of high-throughput sequencing technologies across various pregnancy-related disease studies^20^. Through this comparative analysis of methodologies, our findings not only provide novel insights into the pathophysiology of LETB but also present potential molecular targets for the development of new diagnostic tools and therapeutic strategies, ultimately aiming to improve reproductive health outcomes for affected women.

Future research integrating proteomics, metabolomics, and imaging data with transcriptomics is likely to enhance the resolution and translational relevance of LETB studies. Multi-omics approaches could validate and extend the present findings by correlating transcriptomic changes with protein activity, metabolic shifts, and tissue-level changes observed in radiomics. For example, combining transcriptomic biomarkers with metabolic pathway alterations could yield more reliable diagnostic and prognostic markers. While this study forms a basis for LETB biomarker discovery, incorporating these advanced methodologies would provide a more comprehensive understanding of LETB pathogenesis and improve the development of precise diagnostic and therapeutic strategies.

Building on our findings, future research should focus on expanding sample sizes and integrating proteomic and metabolomic data to provide a more comprehensive understanding of LETB’s impact on infertility. Longitudinal studies are needed to explore the progression of LETB and its direct effects on reproductive outcomes. Additionally, functional studies of the identified biomarkers will help elucidate their roles in LETB pathogenesis and pave the way for the development of targeted diagnostic tools and treatments, ultimately improving fertility outcomes for affected women.

## Conclusion

This study identifies and validates novel inflammatory biomarkers for LETB, offering new insights into its pathogenesis and potential avenues for improved diagnostic and therapeutic strategies. The identified biomarkers, including IFI30, HCK, SPI1, IL1B, ITGB2, and FCGR2A, hold promise for enhancing the early detection and treatment of LETB, thereby improving reproductive health outcomes for affected women.

## Data Availability

Data availability: The sequence data supporting the results of this study has been deposited in the National Center for Biotechnology Information with the main accession number: PRJNA1209661.Persistent web link: http://www.ncbi.nlm.nih.gov/bioproject/1209661.

## References

[1] Chakaya, J. et al. Global tuberculosis report 2020 - reflections on the global TB burden, treatment and prevention efforts. *Int. J. Infect. Dis.* **113** (Suppl 1), 7–S12. 10.1016/j.ijid.2021.02.107 (2021). 33716195 10.1016/j.ijid.2021.02.107 PMC8433257

[2] Ghosh, K., Ghosh, K. & Chowdhury, J. R. Tuberculosis and female reproductive health. *J. Postgrad. Med.* **57**, 307–313. 10.4103/0022-3859.90082 (2011). 22120860 10.4103/0022-3859.90082

[3] Sharma, J. B., Sharma, E., Sharma, S. & Dharmendra, S. Female genital tuberculosis: revisited. *Indian J. Med. Res.* **148**, S71–S83. 10.4103/ijmr.IJMR_648_18 (2018). 30964083 10.4103/ijmr.IJMR_648_18 PMC6469382

[4] Flynn, J. L. & Chan, J. Tuberculosis: latency and reactivation. *Infect. Immun.* **69**, 4195–4201. 10.1128/IAI.69.7.4195-4201.2001 (2001). 11401954 10.1128/IAI.69.7.4195-4201.2001 PMC98451

[5] Houben, R. M. & Dodd, P. J. The global burden of latent tuberculosis infection: a re-estimation using Mathematical Modelling. *PLoS Med.* **13**, e1002152. 10.1371/journal.pmed.1002152 (2016). 27780211 10.1371/journal.pmed.1002152 PMC5079585

[6] MacLean, E. et al. A systematic review of biomarkers to detect active tuberculosis. *Nat. Microbiol.* **4**, 748–758. 10.1038/s41564-019-0380-2 (2019). 30804546 10.1038/s41564-019-0380-2

[7] Wallis, R. S., Peppard, T. & Hermann, D. Month 2 culture status and treatment duration as predictors of recurrence in pulmonary tuberculosis: model validation and update. *PLoS One*. **10**, e0125403. 10.1371/journal.pone.0125403 (2015). 25923700 10.1371/journal.pone.0125403 PMC4414505

[8] Barry, C. E. 3 et al. The spectrum of latent tuberculosis: rethinking the biology and intervention strategies. *Nat. Rev. Microbiol.* **7**, 845–855. 10.1038/nrmicro2236 (2009). 10.1038/nrmicro2236 PMC4144869 19855401

[9] Goletti, D., Petruccioli, E., Joosten, S. A. & Ottenhoff, T. H. Tuberculosis biomarkers: from diagnosis to Protection. *Infect. Dis. Rep.* **8**, 6568. 10.4081/idr.2016.6568 (2016). 27403267 10.4081/idr.2016.6568 PMC4927936

[10] Chen, S., Zhou, Y., Chen, Y. & Gu, J. Fastp: an ultra-fast all-in-one FASTQ preprocessor. *Bioinformatics* **34**, i884–i890. 10.1093/bioinformatics/bty560 (2018). 30423086 10.1093/bioinformatics/bty560 PMC6129281

[11] Kim, D., Langmead, B. & Salzberg, S. L. HISAT: a fast spliced aligner with low memory requirements. *Nat. Methods*. **12**, 357–360. 10.1038/nmeth.3317 (2015). 25751142 10.1038/nmeth.3317 PMC4655817

[12] Pertea, M. et al. StringTie enables improved reconstruction of a transcriptome from RNA-seq reads. *Nat. Biotechnol.* **33**, 290–295. 10.1038/nbt.3122 (2015). 25690850 10.1038/nbt.3122 PMC4643835

[13] Li, B. & Dewey, C. N. RSEM: accurate transcript quantification from RNA-Seq data with or without a reference genome. *BMC Bioinform.* **12**, 323. 10.1186/1471-2105-12-323 (2011). 10.1186/1471-2105-12-323 PMC3163565 21816040

[14] Love, M. I., Huber, W. & Anders, S. Moderated estimation of Fold change and dispersion for RNA-seq data with DESeq2. *Genome Biol.* **15**, 550. 10.1186/s13059-014-0550-8 (2014). 25516281 10.1186/s13059-014-0550-8 PMC4302049

[15] Wang, L., Feng, Z., Wang, X., Wang, X. & Zhang, X. DEGseq: an R package for identifying differentially expressed genes from RNA-seq data. *Bioinformatics* **26**, 136–138. 10.1093/bioinformatics/btp612 (2010). 19855105 10.1093/bioinformatics/btp612

[16] Xie, C. et al. KOBAS 2.0: a web server for annotation and identification of enriched pathways and diseases. *Nucleic Acids Res.* **39**, W316–322. 10.1093/nar/gkr483 (2011). 21715386 10.1093/nar/gkr483 PMC3125809

[17] Kanehisa, M. & Goto, S. KEGG: kyoto encyclopedia of genes and genomes. *Nucleic Acids Res.* **28**, 27–30. 10.1093/nar/28.1.27 (2000). 10592173 10.1093/nar/28.1.27 PMC102409

[18] Kanehisa, M., Furumichi, M., Sato, Y., Kawashima, M. & Ishiguro-Watanabe, M. KEGG for taxonomy-based analysis of pathways and genomes. *Nucleic Acids Res.* **51**, D587–D592. 10.1093/nar/gkac963 (2023). 36300620 10.1093/nar/gkac963 PMC9825424

[19] Kanehisa, M. Toward understanding the origin and evolution of cellular organisms. *Protein Sci.* **28**, 1947–1951. 10.1002/pro.3715 (2019). 31441146 10.1002/pro.3715 PMC6798127

[20] Uuskula, L. et al. Mid-gestational gene expression profile in placenta and link to pregnancy complications. *PLoS One*. **7**, e49248. 10.1371/journal.pone.0049248 (2012). 23145134 10.1371/journal.pone.0049248 PMC3492272

[21] Rull, K. et al. Increased placental expression and maternal serum levels of apoptosis-inducing TRAIL in recurrent miscarriage. *Placenta* **34**, 141–148. 10.1016/j.placenta.2012.11.032 (2013). 23290504 10.1016/j.placenta.2012.11.032 PMC3562443

[22] Panda, S. et al. Identification of differentially recognized T cell epitopes in the spectrum of tuberculosis infection. *Nat. Commun.* **15**, 765. 10.1038/s41467-024-45058-9 (2024). 38278794 10.1038/s41467-024-45058-9 PMC10817963

[23] Mai, D. et al. Exposure to Mycobacterium remodels alveolar macrophages and the early innate response to Mycobacterium tuberculosis infection. *PLoS Pathog*. **20**, e1011871. 10.1371/journal.ppat.1011871 (2024). 38236787 10.1371/journal.ppat.1011871 PMC10796046

[24] Yin, J. et al. Common variants of pro-inflammatory gene IL1B and interactions with PPP1R13L and POLR1G in relation to lung cancer among Northeast Chinese. *Sci. Rep.* **13**, 7352. 10.1038/s41598-023-34069-z (2023). 37147350 10.1038/s41598-023-34069-z PMC10161999

[25] Blazevic, A. et al. Phase 1 open-label dose escalation trial for the development of a human Bacillus Calmette-Guerin Challenge Model for Assessment of tuberculosis immunity in vivo. *J. Infect. Dis.* **229**, 1498–1508. 10.1093/infdis/jiad441 (2024). 38019956 10.1093/infdis/jiad441 PMC11095547

[26] Liu, Y. et al. DNA methylation of ITGB2 contributes to allopurinol hypersensitivity. *Clin. Immunol.* **248**, 109250. 10.1016/j.clim.2023.109250 (2023). 36738816 10.1016/j.clim.2023.109250

[27] Shi, X., Ma, Y., Li, H. & Yu, H. Association between FCGR2A rs1801274 and MUC5B rs35705950 variations and pneumonia susceptibility. *BMC Med. Genet.* **21**, 71. 10.1186/s12881-020-01005-1 (2020). 32252656 10.1186/s12881-020-01005-1 PMC7137230

[28] Lu, S. et al. Fc fragment of immunoglobulin G receptor IIa (FCGR2A) as a new potential prognostic biomarker of esophageal squamous cell carcinoma. *Chin. Med. J. (Engl)*. **135**, 482–484. 10.1097/CM9.0000000000001776 (2021). 34743150 10.1097/CM9.0000000000001776 PMC8869669

[29] Ingale, D. et al. Synovium-synovial fluid Axis in Osteoarthritis Pathology: a Key Regulator of the cartilage degradation process. *Genes (Basel)*. 12. 10.3390/genes12070989 (2021). 10.3390/genes12070989 PMC8305855 34209473

[30] Koks, S., Fernandes, C., Kurrikoff, K., Vasar, E. & Schalkwyk, L. C. Gene expression profiling reveals upregulation of Tlr4 receptors in Cckb receptor deficient mice. *Behav. Brain Res.* **188**, 62–70. 10.1016/j.bbr.2007.10.020 (2008). 18054398 10.1016/j.bbr.2007.10.020

[31] Metsalu, T. et al. Using RNA sequencing for identifying gene imprinting and random monoallelic expression in human placenta. *Epigenetics* **9**, 1397–1409. 10.4161/15592294.2014.970052 (2014). 25437054 10.4161/15592294.2014.970052 PMC4623103

[32] Ma, J., Zhang, X., He, G., Yang, C. & Association between, T. N. F. IL6, IL10 and IFNG polymorphisms and recurrent miscarriage: a case control study. *Reprod. Biol. Endocrinol. 15*. **IL1B**, 83. 10.1186/s12958-017-0300-3 (2017). 10.1186/s12958-017-0300-3 PMC5634870 29017513

[33] Lob, S. et al. Interleukin-1 beta is significantly upregulated in the decidua of spontaneous and recurrent miscarriage placentas. *J. Reprod. Immunol.* **144**, 103283. 10.1016/j.jri.2021.103283 (2021). 33545613 10.1016/j.jri.2021.103283

[34] Zhang, C., Wu, Z., Hu, G., Zhang, Y. & Ao, Z. Exploring characteristics of placental transcriptome and cord serum metabolome associated with low birth weight in Kele pigs. *Trop. Anim. Health Prod.* **55**, 340. 10.1007/s11250-023-03733-x (2023). 37770796 10.1007/s11250-023-03733-x

[35] Wang, Y. X. et al. Downregulation of PDCD4 by deSUMOylation associates with the progression of gestational trophoblastic disease. *Placenta* **130**, 17–24. 10.1016/j.placenta.2022.10.014 (2022). 36370491 10.1016/j.placenta.2022.10.014

[36] Kim, M. S. et al. Differential expression of Extracellular Matrix and Adhesion molecules in fetal-origin amniotic epithelial cells of preeclamptic pregnancy. *PLoS One*. **11**, e0156038. 10.1371/journal.pone.0156038 (2016). 27218821 10.1371/journal.pone.0156038 PMC4878795

[37] Chen, C. P., Chen, P. C., Pan, Y. L. & Hsu, Y. C. Prenatal lipopolysaccharide exposure induces anxiety-like behaviour in male mouse offspring and aberrant glial differentiation of embryonic neural stem cells. *Cell. Mol. Biol. Lett.* **28**, 67. 10.1186/s11658-023-00480-7 (2023). 37592237 10.1186/s11658-023-00480-7 PMC10436442

